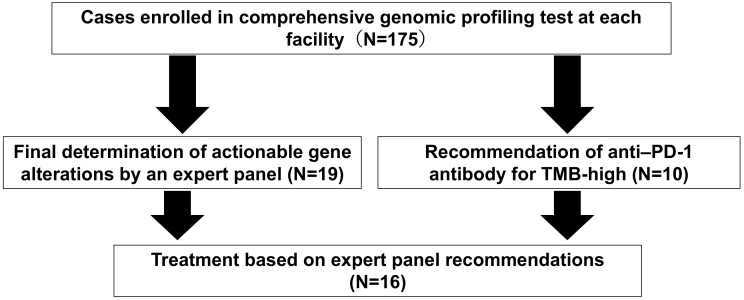# Clinical utility of comprehensive genomic profiling in 175 Japanese patients with advanced skin malignancies refractory to standard therapies

**DOI:** 10.1016/j.jdin.2026.01.012

**Published:** 2026-02-05

**Authors:** Yumi Kambayashi, Taku Fujimura, Hiroshi Kato, Soichiro Kado, Takeo Maekawa, Fumitaka Ono, Takamichi Ito, Megumi Aoki, Shigeto Matsushita, Azusa Miyashita, Satoshi Fukushima, Daiki Rokunohe, Emi Yamazaki, Manami Takahashi-Watanabe, Masayuki Asano, Yoshihide Asano

**Affiliations:** a Department of Dermatology, Tohoku University Graduate School of Medicine, Sendai, Japan; b Department of Geriatric and Environmental Dermatology, Nagoya City University Graduate School of Medical Sciences, Nagoya, Japan; c Department of Dermatology, Jichi Medical University, Shimono, Japan; d Department of Dermatology, Jichi Medical University Saitama Medical Center, Saitama, Japan; e Department of Dermatology, Kyushu University, Fukuoka, Japan; f Department of Dermato-Oncology, NHO Kagoshima Medical Center, Kagoshima, Japan; g Department of Dermatology and Plastic Surgery, Faculty of Life Sciences, Kumamoto University, Kumamoto, Japan; h Department of Dermatology, Hirosaki University, Hirosaki, Japan

**Keywords:** actionable genetic alterations, advanced skin malignancies, comprehensive genomic profiling, TMB

*To the Editor:* Comprehensive genomic profiling (CGP) helps improve diagnostic accuracy and supports individualized therapy for a wide range of cancers.^1^^,^^2^ In skin cancers, particularly melanoma, where therapeutic development has been most advanced,^3^ the identification of actionable gene alterations is expected to drive further innovations in the treatment of advanced skin cancers. We herein contribute real-world evidence from Japanese patients with advanced skin cancers refractory to standard therapies.

In this multicenter retrospective analysis, we evaluated the outcomes of CGP in 175 patients with melanoma, nonmelanoma skin cancers, and cutaneous sarcomas treated at 7 academic institutions in Japan (Table I). Although CGP revealed potentially actionable genomic alterations across tumor types, only 16 patients (9.1%) ultimately received genotype-matched therapy, underscoring the persistent gap between genomic findings and clinical implementation (Supplementary Fig 1, available via Mendeley at https://data.mendeley.com/datasets/nvjyh2tgmn/1). Importantly, immune checkpoint inhibitors were administered to the majority of patients regardless of tumor mutational burden, reflecting current standards of care in melanoma, cutaneous squamous cell carcinoma, and basal cell carcinoma, including post–hedgehog inhibitor settings. In this context, CGP was not intended to replace established first-line treatments, but rather to explore additional therapeutic opportunities in patients with limited remaining options (Table II). Nevertheless, the incremental clinical impact of CGP in this setting remains modest.

**Table I:** Demographics and cancer types of patients undergoing CGP

Age (y)	67 (25-90)
Sex	
Male	99
Female	76
CGP	
F1 CDx	164
F1 liquid CDx	8
Guardant 360	3
Cancer species	
Melanoma	99 (56.6)
NMSC	60 (34.3)
cSCC	22 (12.6)
EMPD	12 (6.9)
Apocrine adenocarcinoma	7 (4.0)
BCC	6 (3.4)
MCC	4 (2.3)
Porpcarcinoma	3 (1.7)
Mucinous carcinoma	1 (0.6)
NUT adnexal carcinoma	1 (0.6)
Sebaceous carcinoma	1 (0.6)
Secretory carcinoma of the skin	1 (0.6)
Meibomian gland carcinoma	1 (0.6)
Adenoid cystic carcinoma	1 (0.6)
Sarcoma	
Angiosarcoma	13 (7.4)
DFSP	2 (1.1)
Leiomyosarcoma	1 (0.6)

Data are reported as median (IQR) or n (%).

*BCC*, Basal cell carcinoma; *CGP*, comprehensive genomic profiling; *cSCC*, cutaneous squamous cell carcinoma; *DFSP*, dermatofibrosarcoma protuberans; *EMPD*, extramammary Paget disease; *F1*, Foundation One; *MCC*, Merkel cell carcinoma; *NMSC*, nonmelanoma skin cancer; *NUT*, nuclear protein of testis.

**Table II:** Summary of the results of comprehensive genomic profiling and matched therapies received

	Age (y)	Sex	Cancer species	TMB	Actionable gene alterations	Recommendation	Received matched therapy	Efficacies
1	62	F	Melanoma	Low	*KIT* p.P551R	Imatinib	Imatinib	PD
2	76	F	Melanoma	Low	*KIT* amplification *BRCA1* p.W1718C	Imatinib PARP inhibitor	ー	ー
3	67	M	Melanoma	Low	*BRAF* p.V600R	BRAF + MEK inhibitor	Dabrafenib + Trametinib	PR
4	60	M	Melanoma	Low	*KIT* p.Y578S	Pembrolizumab + Imatinib	ー	ー
5	73	M	Melanoma	Low	*KIT* p.K642E	Pembrolizumab + Imatinib	ー	ー
6	71	F	Melanoma	Low	*KIT* p.V559D	Pembrolizumab + Imatinib	ー	ー
7	67	M	Melanoma	Low	*BRAF* p.N581S	BRAF + MEK inhibitor	Dabrafenib + Trametinib	SD
8	66	M	Melanoma	High	*KIT* p.A829P *KIT* amplification	Pembrolizumab + Imatinib	Pembrolizumab + Imatinib	PD
9	78	M	Melanoma	Low	*KIT* p.D8230V	Pembrolizumab + Imatinib	ー	ー
10	73	F	Melanoma	Low	*KIT* p.K462E *KIT* amplification	Pembrolizumab + Imatinib	Pembrolizumab + Imatinib	PR
11	73	F	Melanoma	Low	*ZKSCAN5-BRAF* fusion	BRAF + MEK inhibitor	Dabrafenib + Trametinib	PR
12	68	F	Melanoma	Low	*KIT* p.D579del	Pembrolizumab + Imatinib	ー	ー
13	66	F	Angiosarcoma	Low	*PEAR1-NTRK1* fusion	NTRK inhibitor	Larotrectinib sulfate	ー
14	76	M	Angiosarcoma	High	*BCRA2* p.W2626∗	Platinum-based anticancer agents Pembrolizumab	Pembrolizumab	SD
15	60	M	Apocrine adenocarcinoma	Low	*BRCA2* loss	Platinum-based anticancer agents PARP inhibitor	Olaparib	PD
16	60	F	BCC	Low	*BRCA1* p.L63	Platinum-based anticancer agents PARP inhibitor	CDDP + 5-FU	PR
17	89	F	BCC	High	*ー*	Pembrolizumab	Pembrolizumab	PR
18	73	M	EMPD	Low	*ERBB2* p.I767M	Trastuzumab	Trastuzumab + DOC	ー
19	71	F	EMPD	High	*ー*	Pembrolizumab	Pembrolizumab	PD
20	74	F	EMPD	High	*ー*	Pembrolizumab	ー	ー
21	79	M	EMPD	High	*ー*	Pembrolizumab	Pembrolizumab	SD
22	64	M	SCC	High	*ー*	Pembrolizumab	ー	ー
23	90	F	SCC	High	*BRCA1* p.L63∗	Pembrolizumab Platinum-based anticancer agents	Pembrolizumab	CR
24	45	M	SCC	High	*ー*	Pembrolizumab	Pembrolizumab	CR
25	75	M	SCC	High	*ー*	Pembrolizumab	ー	ー
26	43	M	Secretory carcinoma	Low	*ETV6-NTRK3* fusion	NTRK inhibitor	ー	ー

*BCC*, Basal cell carcinoma; *CDDP*, cisplatin; *CR,* complete responce; *DOC*, docetaxel; *EMPD*, extramammary Paget disease; *F*, female; *5-FU*: fluorouracil; *M*, male; *NTRK*, neurotrophic tyrosine receptor kinase; *PARP*, poly(ADP-ribose)polymerase; *PD,* progress disease; *PR,* partial response; *SCC*, squamous cell carcinoma; *SD*, stable disease; *TMB*, tumor mutation burden.

Actionable alterations identified in our cohort included non-V600 BRAF mutations, KIT alterations, BRCA1/2 loss, ERBB2 mutations, and NTRK fusions. Among patients with melanoma who received matched therapy, responses were observed in selected cases treated with BRAF/MEK inhibitors or KIT-directed therapies, consistent with prior reports.^4^ In nonmelanoma skin cancer, particularly cutaneous squamous cell carcinoma and basal cell carcinoma, high tumor mutational burden emerged as the most actionable biomarker, with pembrolizumab achieving durable responses in several patients (Supplementary Table I, available via Mendeley at https://data.mendeley.com/datasets/nvjyh2tgmn/1). These findings suggest that, at present, CGP may have greater practical value for identifying immunotherapy-eligible patients than for enabling widespread targeted therapy in cutaneous malignancies.

The low adoption rate of expert panel recommendations warrants particular attention. Several factors likely contributed, including rapid disease progression, limited access to genotype-matched clinical trials, regulatory and reimbursement barriers for off-label targeted agents, and clinician preference for established therapies with known efficacy. In addition, institutional differences in whether recommendations were issued for alterations without trial availability influenced the absolute number of actionable findings, although the proportion of patients ultimately treated remained consistently low across centers. Taken together, our findings indicate that the current clinical utility of CGP in advanced skin malignancies is limited, particularly outside of immunotherapy selection. Rather than demonstrating definitive outcome improvements, this study highlights real-world barriers that hinder the translation of genomic data into patient benefit. Expansion of clinical trial infrastructure, improved access to targeted agents, and earlier integration of CGP into the treatment timeline may be required to fully realize its potential in dermatologic oncology. We believe these observations are relevant to clinicians considering CGP in routine practice and underscore the need for systemic solutions beyond sequencing itself.

### Data availability statement

The data that support the findings of this study are available on request from the corresponding author.

## Conflicts of interest

None disclosed.

## Figures and Tables

**Supplementary Fig 1:**